# Injury Patterns and Physiologic Risk Stratification in Facial Trauma Patients with Orbital Fractures: A National Trauma Database Analysis

**DOI:** 10.3390/cmtr18040052

**Published:** 2025-12-06

**Authors:** Turki Bin Mahfoz

**Affiliations:** Department of Otolaryngology, Head and Neck Surgery, Faculty of Medicine, Imam Mohammad Ibn Saud Islamic University (IMSIU), Riyadh 13317-4233, Saudi Arabia; tmmahfoz@imamu.edu.sa

**Keywords:** orbital fractures, risk stratification, injury mechanisms, trauma registry, age factors

## Abstract

Background: Although orbital fractures are common in trauma care, age-specific mechanisms and admission physiology-based risk stratification have not been systematically characterized. This study aimed to identify age–mechanism interaction patterns and develop an admission-based physiological risk score for orbital fracture patients. Methods: This retrospective cohort study analyzed 41,464 adult orbital fracture patients from the National Trauma Data Bank (2018–2020). A three-component physiological risk score was developed using admission vital signs: severe hypotension (<90 mmHg, 2 points), tachycardia (>100 bpm, 1 point), and severe traumatic brain injury (GCS ≤ 8, 1 point). Risk stratification performance was validated against composite adverse outcomes. Results: Distinct age–mechanism patterns emerged: 74.0% of elderly patients (≥65 years) sustained falls, while young adults demonstrated a bimodal distribution with motor vehicle crashes (31.2%) and violence (28.4%). Violence-related injuries occurred in younger patients (40.3 vs. 55.0 years) but had lower injury severity scores (10.0 vs. 14.4) and mortality (2.8% vs. 5.2%) than accidental mechanisms. High-/critical-risk patients (8.4% of the cohort) had 16.2% mortality versus 2.1% in stable patients. Complex facial injuries demonstrated 11-fold higher mortality (7.7% vs. 0.7%). The physiologic risk score achieved AUC 0.79 (95% CI: 0.78–0.80). Conclusions: Age–mechanism interactions revealed distinct bimodal injury patterns in young adults. Admission physiologic parameters effectively identify 8.4% of patients requiring intensive resources, while violence-related injuries paradoxically demonstrate better outcomes than accidental mechanisms.

## 1. Introduction

Orbital fractures represent a predominant category of facial injuries encountered in trauma centers globally, constituting 4–16% of all facial fractures with significant variation across geographic regions and healthcare systems [1]. These injuries encompass diverse traumatic mechanisms, from low-energy falls in elderly populations to high-velocity collisions in younger demographics, generating substantial heterogeneity in injury patterns and clinical outcomes.

Current orbital fracture management relies predominantly on radiographic imaging and clinical examination, with insufficient integration of admission physiological markers as predictive indicators [2]. While injury severity scoring systems provide comprehensive trauma assessment frameworks, orbital fracture patients frequently present with complex concomitant facial and cranial injuries inadequately quantified through conventional methodologies. Previous studies have demonstrated the potential of standardized assessment tools, with Yadav and Haukoos developing clinical risk scores for traumatic orbital fractures [3]. Existing orbital fracture risk models by Basta and Scolozzi have focused on guiding surgical decisions for definitive management [4,5]. However, tools for emergency department triage and resource allocation using admission physiology remain limited.

The convergence of patient demographics and injury mechanisms represents a critical knowledge deficit with significant implications for care delivery and prevention strategies. Age-related variations in injury mechanisms have been documented in general trauma populations; however, specific patterns in orbital fracture patients remain inadequately analyzed. Shome identified distinct facial fracture patterns across age groups [1], while Lin demonstrated significant variations in orbital involvement across maxillofacial fracture patients [6]. Violence-related orbital injuries constitute a particularly significant subgroup [7,8], with Mancera demonstrating associations with racial segregation and neighborhood-level social determinants [9], and Arpalahti revealing significant psychiatric disorder associations requiring comprehensive psychosocial assessment [10].

The primary objectives of this study were to characterize age–mechanism interaction patterns in orbital fracture patients, develop a physiological risk stratification score utilizing admission parameters, and compare clinical outcomes between violence-related and accidental orbital fractures.

## 2. Methods

This retrospective cohort study analyzed data from the National Trauma Data Bank (NTDB) spanning a three-year period from 2018 to 2020. Adult patients with orbital fractures were identified from the NTDB registry during the study period. The inclusion criteria were age ≥16 years and documented orbital fracture diagnosis. The exclusion criteria were age < 16 years, interfacility transfers, missing patient identifiers, and invalid injury severity scores (ISS < 1 or >75). After applying the inclusion and exclusion criteria, 41,464 adult patients comprised the final analytic cohort.

Age was analyzed as a continuous variable and stratified into three categories: young (<35 years), middle-aged (35–64 years), and elderly (≥65 years). Five injury mechanism categories were analyzed: falls, motor vehicle crashes, violence, being struck by objects, and other/unknown mechanisms. Injury severity was assessed using the Injury Severity Score (ISS) and the Glasgow Coma Scale (GCS). The Abbreviated Injury Scale (AIS) was used to classify facial and head injury severities.

A three-component physiological risk score was developed from admission parameters [11,12,13,14]. Points were assigned as follows: 2 points for severe hypotension (systolic blood pressure < 90 mmHg), 1 point for tachycardia (pulse rate > 100 bpm), and 1 point for severe traumatic brain injury (GCS ≤ 8). Based on the cumulative score, patients were categorized as stable (0 points), moderate risk (1 point), or high/critical risk (≥2 points). Facial injury complexity was dichotomized as simple (no severe facial injury and no concurrent head injury) or complex (severe facial injury AIS ≥ 3 or concurrent head injury AIS ≥ 2).

The primary composite adverse outcome was defined as the occurrence of any of the following: ICU admission, in-hospital mortality, major complications, or emergency procedures. Major complications were defined according to National Trauma Data Bank standardized criteria and included acute respiratory distress syndrome, pneumonia, sepsis, acute kidney injury requiring dialysis, cardiac arrest, pulmonary embolism, deep vein thrombosis, unplanned return to the operating room, and wound dehiscence. Emergency procedures were defined as any urgent surgical or interventional procedure performed within 24 h of emergency department arrival, including craniotomy, exploratory laparotomy, thoracotomy, fasciotomy, external fixation, damage control surgery, and emergent intubation. Continuous variables were expressed as means with standard deviations and compared using analysis of variance (ANOVA). Categorical variables were expressed as frequencies with percentages and compared using the chi-square test. Statistical significance was set at *p* < 0.05. Receiver operating characteristic (ROC) curve analysis was performed to evaluate the discriminative ability of the physiologic risk score for predicting adverse outcomes. The area under the curve (AUC) with 95% confidence intervals was calculated for each outcome using the DeLong method. Optimal cutpoints were determined using Youden’s index (maximum sensitivity + specificity − 1). All analyses were performed using R version 4.3.0 (R Foundation for Statistical Computing, Vienna, Austria) with the pROC package.

## 3. Results

### 3.1. Cohort Characteristics and Injury Mechanisms

During the study period (2018–2020), 41,464 adult patients with orbital fractures met the inclusion criteria. Falls represented the leading mechanism (*n* = 13,048, 31.5%), followed by violence (*n* = 9297, 22.4%), motor vehicle crashes (*n* = 7885, 19.0%), other/unknown mechanisms (*n* = 10,111, 24.4%), and being struck by an object (*n* = 1123, 2.7%). Overall, 29,219 patients (70.5%) were male, with a mean age of 49.8 ± 19.7 years.

**Figure 1:**
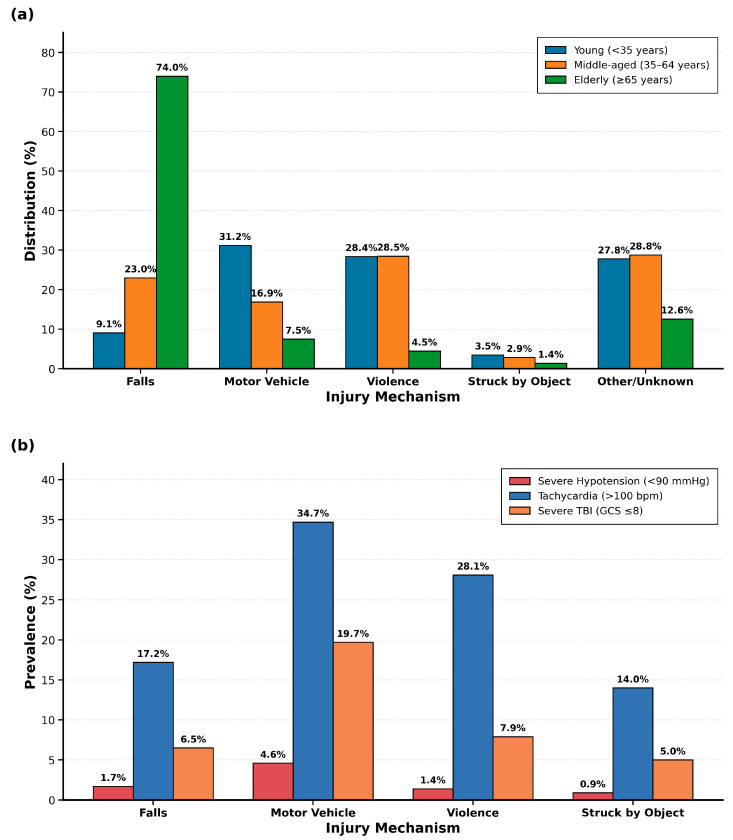
(**a**) Age Distribution Patterns by Mechanism of Injury Grouped bar chart showing percentage distribution of three adult age groups (young < 35 years, middle-aged 35–64 years, elderly ≥ 65 years) across five injury mechanisms. Age-specific injury patterns were demonstrated, with elderly patients predominantly sustaining falls (74.0%), young adults predominantly sustaining motor vehicle crashes (31.2%) and violence (28.4%), and middle-aged patients showing violence as the leading mechanism (28.5%). *p* < 0.001 for age–mechanism interaction. (**b**) Physiological Risk Marker Prevalence by Mechanism of Injury Grouped bar chart showing prevalence of three physiologic risk markers (severe hypotension < 90 mmHg, tachycardia > 100 bpm, severe TBI GCS ≤ 8) across five injury mechanisms. Motor vehicle crashes and other/unknown mechanisms demonstrate highest rates of tachycardia (34.7% and 32.3%) and severe TBI (19.7% and 23.5%), while falls show lowest rates across all physiologic markers. *p* < 0.001 for all comparisons.

Distinct age–mechanism interaction patterns emerged across the study population (Figure 1a, *p* < 0.001). Elderly patients (≥65 years, *n* = 10,459, 25.2%) sustained predominantly falls (74.0%), reflecting age-related balance deterioration, polypharmacy effects, and environmental hazards. Young adults (<35 years, *n* = 13,038, 31.4%) exhibited a bimodal distribution between motor vehicle crashes (31.2%) and violence (28.4%), suggesting that prevention strategies must address both traffic safety and violence intervention. Middle-aged patients (35–64 years, *n* = 17,967, 43.3%) showed violence as the predominant mechanism (28.5%), indicating that interpersonal violence risk extends throughout middle adulthood with implications for emergency screening protocols.

The mean patient age varied significantly by mechanism: falls 65.1 ± 18.1 years, motor vehicle crashes 38.3 ± 17.3 years, violence 40.3 ± 14.0 years, struck by object 42.4 ± 18.6 years, and other/unknown 43.8 ± 17.1 years (*p* < 0.001). Male representation was highest in violence-related injuries (84.3%) and lowest in fall-related injuries (54.3%) (*p* < 0.001).

Critical physiological derangements showed mechanism-specific patterns (Figure 1b) with direct triage implications. Motor vehicle crashes demonstrated the highest rates of tachycardia (34.7%) and severe TBI with GCS ≤ 8 (19.7%), indicating need for immediate intensive monitoring and neurosurgical consultation. Other/unknown mechanisms showed similarly elevated rates (tachycardia 32.3%, severe TBI 23.5%), suggesting high-energy trauma requiring aggressive resuscitation. Falls showed the lowest prevalence of all physiologic markers (severe hypotension 1.7%, tachycardia 17.2%, severe TBI 6.5%), reflecting different pathophysiology where morbidity derives from baseline frailty and delayed complications rather than immediate physiologic derangement. Violence-related injuries occupied an intermediate position (tachycardia 28.1%, severe hypotension 1.4%), supporting that interpersonal violence produces more localized facial trauma without multi-system injury patterns, allowing for observation units rather than intensive monitoring in isolated injuries. The prevalence of severe TBI ranged from 6.5% (falls) to 23.5% (other/unknown), emphasizing the importance of high clinical suspicion for intracranial pathology in motor vehicle crashes and high-energy mechanisms.

### 3.2. Injury Severity and Physiologic Assessment

Injury severity scores differed significantly across mechanisms (*p* < 0.001). Motor vehicle crashes and other/unknown mechanisms demonstrated the highest severity (18.0 ± 11.0 and 18.6 ± 11.4, respectively), while violence and struck by object injuries had lower scores (both 10.0 ± 7.4–7.8). Glasgow Coma Scale scores were lowest for other/unknown mechanisms (11.9 ± 4.6) and motor vehicle crashes (12.3 ± 4.4) compared to other mechanisms (13.8–14.2, *p* < 0.001).

Critical physiological derangements showed mechanism-specific patterns (Figure 1b). Severe hypotension (<90 mmHg) was more frequent in other/unknown mechanisms (5.0%) and motor vehicle crashes (4.6%) than in violence (1.4%) and falls (1.7%) (*p* < 0.001). Tachycardia (>100 bpm) occurred most commonly in motor vehicle crashes (34.7%) and least frequently in falls (17.2%) (*p* < 0.001). Severe traumatic brain injury (GCS ≤ 8) was present in 23.5% of other/unknown mechanisms and 19.7% of motor vehicle accidents (*p* < 0.001).

Compared to stable patients, those in the high-/critical-risk category had 4.2-fold higher odds of polytrauma (OR 4.21, 95% CI: 3.92–4.52) and 5.3-fold higher odds of concurrent head injury (OR 5.34, 95% CI: 4.89–5.82). The odds of severe facial injury increased progressively across risk categories, with moderate-risk patients showing 1.7-fold higher odds (OR 1.74, 95% CI: 1.52–2.00) and high-/critical-risk patients showing 3.5-fold higher odds (OR 3.48, 95% CI: 2.96–4.09) compared to stable patients (Table 1).

### 3.3. Physiologic Risk Score Performance

The physiological risk score stratified patients into three categories (Table 2). Stable patients (0 points, n = 27,134, 65.4%), moderate-risk patients (1 point, n = 10,842, 26.1%), and high-/critical-risk patients (≥2 points, n = 3488, 8.4%) differed significantly in demographics. High-/critical-risk patients were younger (42.4 ± 17.9 vs. 51.5 ± 20.8 years, *p* < 0.001) with higher male representation (76.3% vs. 68.6%, *p* < 0.001). Injury severity scores differed across risk categories: high-/critical-risk patients (27.1 ± 13.6), moderate risk (16.2 ± 11.0), and stable patients (11.9 ± 7.6) (*p* < 0.001). The maximum head injury severity and total injury burden also differed significantly across the groups (*p* < 0.001).

Polytrauma was present in 32.9% of stable patients, 46.0% of moderate-risk patients, and 67.4% of high-/critical-risk patients (*p* < 0.001). Concurrent head injury (AIS score ≥ 2) was present in 48.4% of stable patients and 83.3% of high-/critical-risk patients (*p* < 0.001).

### 3.4. Facial Injury Complexity and Clinical Outcomes

Complex facial injuries (severe facial injury AIS ≥ 3 or concurrent head injury AIS ≥ 2) affected 22,593 patients (54.5%) and were associated with substantially different clinical trajectories than simple injuries (Table 2). ICU admission rates were markedly higher in complex injuries (58.2%) than in simple injuries (16.9%) (*p* < 0.001). In-hospital mortality demonstrated a pronounced difference, with complex injuries having a mortality rate of 7.7% compared with 0.7% for simple injuries (*p* < 0.001). Resource utilization patterns revealed significant differences between the complexity groups. ICU length of stay was longer for complex injuries (7.4 ± 9.0 days) than for simple injuries (4.9 ± 6.2 days) (*p* < 0.001). Major procedure requirements were higher in complex injuries (0.9 ± 1.2) than in simple injuries (0.3 ± 0.7) (*p* < 0.001). Emergency procedures were performed in 63.8% of complex injuries compared to 61.7% of simple injuries (*p* < 0.001), while complication rates were identical between groups (32.0% vs. 32.0%, *p* = 0.989). The composite adverse outcome rate was 71.9% for complex injuries and 43.4% for simple injuries (*p* < 0.001).

Complex facial injuries demonstrated substantially elevated odds of adverse outcomes compared to simple injuries. The odds of in-hospital mortality were nearly 12-fold higher (OR 11.97, 95% CI: 10.01–14.32), while ICU admission odds were approximately 7-fold higher (OR 6.87, 95% CI: 6.58–7.18). The composite adverse outcome occurred with over 3-fold higher odds in complex injuries (OR 3.33, 95% CI: 3.20–3.47). Notably, complication rates were identical between groups (OR 1.00, 95% CI: 0.96–1.04, *p* = 0.989), suggesting that complexity primarily affects mortality and resource utilization rather than overall complication frequency.

### 3.5. Violence-Related Injury Characteristics

Violence-related orbital fractures (*n* = 9297, 22.4%) demonstrated distinct epidemiologic and clinical characteristics compared to accidental injuries (falls and motor vehicle crashes combined, *n* = 20,933) (Table 3). Violence-related injured patients were significantly younger (40.3 ± 14.0 vs. 55.0 ± 19.2 years, *p* < 0.001) and showed marked male predominance (84.3% vs. 58.8%, *p* < 0.001). Violence-related injuries had lower injury severity scores (10.0 ± 7.4 vs. 14.4 ± 9.7, *p* < 0.001).

### 3.6. Risk Score Validation

The physiologic risk score demonstrated excellent discrimination for in-hospital mortality (AUC 0.809, 95% CI 0.799–0.819) and good discrimination for the composite outcome of ICU admission or death (AUC 0.662, 95% CI 0.657–0.666). ICU admission alone showed similar performance (AUC 0.659, 95% CI 0.655–0.664). At the optimal cutpoint of ≥1, the score achieved 84.5% sensitivity and 98.9% negative predictive value for mortality, making it highly effective for ruling out high-risk patients. For the composite outcome of ICU admission or death, a cutpoint of ≥1 yielded 52.0% sensitivity, 76.9% specificity, 59.7% positive predictive value, and 70.9% negative predictive value. Risk stratification analysis revealed that patients with a score of 0 had a 4.1% mortality rate, which increased to 11.0% for score ≥ 1, 24.4% for score ≥ 2, and 35.9% for score ≥ 3. The composite outcome rate similarly increased from 39.7% (score 0) to 87.3% (score ≥ 2). The score showed no discrimination for postoperative complications (AUC 0.497, 95% CI 0.492–0.502).

## 4. Discussion

This retrospective analysis of traumatic orbital fracture patients revealed clinically significant findings. The identification of distinct age–mechanism interaction patterns and the development of a validated physiological risk stratification system offer novel insights for both targeted injury prevention initiatives and acute care decision-making protocols.

The pronounced age–mechanism interaction patterns observed in this investigation demonstrate that orbital fracture epidemiology exhibits fundamental differences across demographic groups, with substantial implications for the development of targeted prevention strategies [7,8]. The finding that most elderly patients sustained falls while young adults demonstrated a bimodal distribution between motor vehicle crashes and violence indicates that prevention efforts must be age-specific rather than mechanism-agnostic. The predominance of falls in elderly populations aligns with established age-related physiological changes, including balance deterioration, decreased bone density, and polypharmacy effects, while the bimodal pattern in young adults reflects their differential exposure to both high-energy vehicular trauma and interpersonal violence mechanisms [15].

The injury pattern of the middle-aged demographic group, characterized by violence as the predominant mechanism, suggests that interpersonal violence remains a significant risk factor throughout adulthood rather than being confined to younger populations. This epidemiologic finding carries important implications for emergency department screening protocols and social service intervention strategies, as violence-related orbital fractures may function as sentinel injuries, indicating ongoing domestic or interpersonal violence exposure requiring comprehensive safety assessment and intervention planning.

The most striking finding in this investigation involves the pattern observed in violence-related orbital fractures, which demonstrated consistently superior clinical outcomes compared to accidental injuries, despite affecting a younger, predominantly male population [9,16]. Violence-related injuries exhibited lower injury severity scores, reduced ICU admission rates, and significantly lower mortality compared to the combined accidental injury group encompassing falls and motor vehicle crashes.

This finding may reflect several important clinical and mechanistic considerations underlying the pathophysiology of the injury. Violence-related facial injuries typically involve more localized trauma patterns and demonstrate a reduced likelihood of high-energy mechanisms that precipitate multi-system traumatic injuries. Conversely, the accidental injury cohort included high-energy motor vehicle crashes and falls from significant heights in elderly patients with multiple comorbidities, both clinical scenarios associated with severe polytrauma presentations. The lower concurrent head injury rates observed in violence-related injuries support this mechanistic hypothesis, suggesting that interpersonal violence characteristically results in more isolated facial trauma rather than the complex multi-system injury patterns observed in vehicular crashes or significant falls.

The development and validation of the three-component physiological risk stratification score addresses a critical knowledge gap in emergency orbital fracture management protocols [17]. The demonstrated ability to identify 8.4% of patients requiring intensive care resources utilizing simple admission parameters (blood pressure, heart rate, and Glasgow Coma Scale) provides immediate clinical utility in high-volume emergency departments. High-/critical-risk patients demonstrated dramatically different clinical trajectories, with higher proportion requiring ICU admission.

The moderate discriminative capability of the risk stratification score (AUC 0.79) represents a clinically meaningful performance for emergency triage applications, particularly considering its simplicity and reliance on readily available admission assessment data [18,19]. The score’s implementation requires only vital signs and Glasgow Coma Scale assessment available within minutes of patient arrival, making it feasible for diverse clinical settings including resource-limited environments. In settings with limited access to advanced imaging or subspecialty services, the risk score can guide critical transfer decisions to higher-level trauma centers, ensuring that high-/critical-risk patients reach appropriate facilities for definitive care. Early risk stratification facilitates appropriate multidisciplinary care pathway activation, including ophthalmology consultation for high-risk patients, neurosurgical evaluation when severe TBI is present, and critical care involvement for patients requiring intensive monitoring. The identified age–mechanism interaction patterns can inform clinical suspicion indices and guide appropriate imaging protocols and subspecialty consultation decisions. Of note, our risk score uses standard ATLS physiological markers (blood pressure, heart rate, GCS) from routine ABCDE assessment.

The finding that complex facial injuries demonstrated an association with higher mortality carries significant implications for trauma center resource allocation and staffing decision algorithms [20,21]. The substantial increase in ICU length of stay and major procedure requirements in complex injury presentations suggests that facial injury complexity assessment should be incorporated into early resource planning protocols and capacity management systems. Notably, while overall complication rates remained identical between simple and complex injuries, the types and severity of complications likely differ substantially, with complex injuries requiring more intensive therapeutic interventions despite similar complication frequencies [22]. This epidemiologic pattern suggests that complexity classification based on Abbreviated Injury Scale scores and concurrent head injury assessment effectively identifies patient populations requiring specialized care pathways and multidisciplinary management approaches.

These research findings demonstrate immediate applications for emergency department protocols and trauma team activation criteria optimization [23,24]. The physiologic risk score demonstrated robust discriminative ability for mortality and ICU admission, with performance comparable to established trauma scoring systems but using only three readily available variables. The excellent mortality prediction and high negative predictive value at the optimal cutpoint support the score’s utility for early risk stratification and triage decisions. The good discrimination for ICU admission or death provides a clinically actionable composite outcome for resource allocation. The absence of discrimination for postoperative complications is noteworthy and suggests that complications in orbital trauma are primarily determined by surgical technique, injury complexity, and procedural factors rather than initial physiologic status. This finding has important implications for quality improvement initiatives, suggesting that complication reduction efforts should focus on surgical processes rather than patient selection. The simplicity of the score (three objective variables routinely collected on presentation) enhances its feasibility for implementation in emergency and trauma settings without requiring additional data collection or complex calculations. The clear risk thresholds identified (score 0 for low risk, score 1 for moderate risk requiring enhanced monitoring, and score ≥ 2 for high risk requiring direct ICU admission) provide actionable clinical decision points that can be integrated into institutional protocols.

The physiological risk stratification score can be calculated within minutes of patient arrival using standard vital sign assessment and neurologic evaluation, facilitating early identification of patients requiring intensive monitoring and intervention. The proposed risk score incorporates physiological parameters routinely obtained during initial Advanced Trauma Life Support assessment, specifically systolic blood pressure, heart rate, and Glasgow Coma Scale. While these parameters are established markers of physiological decompensation in polytrauma, their specific prognostic value and optimal weighting for risk stratification in orbital fracture patients have not been previously validated. The present study demonstrates that a weighted scoring system based on these admission physiological variables achieves moderate discriminatory ability for predicting adverse outcomes, with patients classified as high risk exhibiting substantially elevated mortality compared to physiologically stable patients. This represents the first validation, to our knowledge, of a quantitative risk stratification tool based solely on admission physiology in this specific patient population, potentially facilitating objective triage decisions in the acute setting.

This investigation demonstrates several limitations inherent to large trauma registry analyses that warrant consideration in the interpretation of the results. The retrospective design precludes definitive causality assessment, and reliance on administrative coding systems may introduce classification bias, affecting result precision. The study period (2018–2020) encompasses the initial phase of the COVID-19 pandemic, which may have had minimal influence on injury patterns during 2020. However, this represents only one-third of our study period, with the majority of data collected during pre-pandemic years, suggesting limited impact on the overall findings. Additionally, the National Trauma Data Bank lacks detailed information regarding injury circumstances, perpetrator relationships in violence cases, and long-term functional outcome assessments.

The NTDB does not consistently capture detailed ophthalmologic examination findings including visual acuity, diplopia, and extraocular movements, limiting our ability to include orbital-specific functional parameters in the risk score. Future prospective studies should incorporate these clinically relevant measures to develop more comprehensive orbital fracture-specific risk stratification tools.

Patients with missing values for any of the three physiological risk score components (systolic blood pressure, heart rate, or Glasgow Coma Scale) were excluded from risk stratification analyses but included in descriptive and epidemiologic analyses to maximize sample size. The relatively low missingness rates and consistent findings across analytical approaches support the validity of our conclusions. Future prospective studies with standardized data capture protocols would further strengthen these findings.

Important clinical factors not consistently captured in NTDB include patient comorbidities, anticoagulant use, frailty indices, and pre-injury functional status. These factors, particularly relevant in elderly patients with fall-related injuries, may significantly influence outcomes and should be incorporated in future prospective studies. External validation in independent patient cohorts is required before clinical implementation. Future studies should validate the risk score across different trauma systems and healthcare settings.

## 5. Conclusions

This large-scale analysis demonstrates that orbital fracture epidemiology is characterized by distinct age–mechanism interactions that have not been previously well characterized. The development of a simple, validated physiological risk score provides emergency clinicians with an immediate tool for identifying the minority of patients who will require intensive resources. This simple, objective scoring system provides clinically actionable risk stratification using only three routinely collected variables, supporting its implementation for triage and ICU resource allocation in orbital trauma care. These findings support the implementation of age-specific prevention strategies and evidence-based risk stratification protocols for orbital fracture management.

## Figures and Tables

**Table 1:** Injury Characteristics by Physiologic Risk Category.

Variable	Stable (n = 27,134)	Moderate Risk (n = 10,842)	OR (95% CI) ^a^	High/Critical Risk (n = 3488)	OR (95% CI) ^a^	*p*-Value
**Demographics**						
**Age, years, mean (SD)**	51.5 (20.8)	43.5 (18.4)	-	42.4 (17.9)	-	<0.001
**Male sex, n (%)**	18,626 (68.6)	7932 (73.2)	1.25 (1.19–1.31)	2661 (76.3)	1.47 (1.34–1.61)	<0.001
**Injury Severity Measures**						
**Injury Severity Score, mean (SD)**	11.9 (7.6)	16.2 (11.0)	-	27.1 (13.6)	-	<0.001
**Maximum AIS Face, mean (SD)**	2.0 (0.2)	2.0 (0.2)	-	2.1 (0.3)	-	<0.001
**Maximum AIS Head, mean (SD)**	1.4 (1.5)	1.9 (1.8)	-	3.2 (1.7)	-	<0.001
**Total injuries, mean (SD)**	6.9 (4.0)	8.9 (5.5)	-	13.6 (6.8)	-	<0.001
**Injury Patterns**						
**Polytrauma, n (%)**	8938 (32.9)	4987 (46.0)	1.74 (1.67–1.81)	2351 (67.4)	4.21 (3.92–4.52)	<0.001
**Multiple body regions, n (%)**	337 (1.2)	159 (1.5)	1.19 (0.98–1.44)	65 (1.9)	1.51 (1.16–1.98)	0.006
**Severe facial injury (AIS ≥ 3), n (%)**	528 (1.9)	362 (3.3)	1.74 (1.52–2.00)	225 (6.5)	3.48 (2.96–4.09)	<0.001
**Concurrent head injury (AIS ≥ 2), n (%)**	13,128 (48.4)	6309 (58.2)	1.49 (1.43–1.55)	2907 (83.3)	5.34 (4.89–5.82)	<0.001

^a^ Odds ratios with 95% confidence intervals comparing Moderate Risk and High/Critical Risk groups to Stable (reference) group.

**Table 2:** Clinical Outcomes by Facial Injury Complexity.

Variable	Simple (n = 18,871)	Complex (n = 22,593)	OR (95% CI) ^a^	*p*-Value
**Resource Utilization**				
**ICU length of stay, days, mean (SD)**	4.9 (6.2)	7.4 (9.0)		<0.001
**Major procedure count, mean (SD)**	0.3 (0.7)	0.9 (1.2)		<0.001
**Clinical Outcomes**				
**ICU admission, n (%)**	3180 (16.9)	13,159 (58.2)	6.87 (6.58–7.18)	<0.001
**In-hospital mortality, n (%)**	130 (0.7)	1733 (7.7)	11.97 (10.01–14.32)	<0.001
**Any complications, n (%)**	6042 (32.0)	7241 (32.0)	1.00 (0.96–1.04)	0.989
**Emergency procedure, n (%)**	11,635 (61.7)	14,414 (63.8)	1.09 (1.05–1.14)	<0.001
**Composite adverse outcome, n (%)**	8196 (43.4)	16,241 (71.9)	3.33 (3.20–3.47)	<0.001

^a^ Odds ratios with 95% confidence intervals comparing Complex to Simple (reference) group.

**Table 3:** Comparison of Violence-Related versus Accidental Orbital Fractures.

Variable	Accidental ^1^ (n = 20,933)	Violence-Related (n = 9297)	*p*-Value
**Demographics**			
Age, years, mean (SD)	55.0 (19.2)	40.3 (14.0)	<0.001
Male sex, n (%)	12,307 (58.8)	7838 (84.3)	<0.001
**Injury Characteristics**			
Injury Severity Score, mean (SD)	14.4 (9.7)	10.0 (7.4)	<0.001
Severe facial injury (AIS ≥ 3), n (%)	464 (2.2)	177 (1.9)	0.118

^1^ Accidental includes falls and motor vehicle crashes.

## Data Availability

Data supporting the findings of this study are available from the American College of Surgeons National Trauma Data Bank. Restrictions apply to the availability of these data, which were used under license for the current study and, thus, are not publicly available. However, the data are available from the authors upon reasonable request and with permission from the American College of Surgeons.
